# Coffee consumption and diabetic retinopathy in adults with diabetes mellitus

**DOI:** 10.1038/s41598-022-07192-6

**Published:** 2022-03-03

**Authors:** Hak Jun Lee, Ji In Park, Sung Ok Kwon, Daniel Duck-Jin Hwang

**Affiliations:** 1 Department of Ophthalmology, Hangil Eye Hospital, Incheon, 21388 Korea; 2 Department of Medicine, Kangwon National University Hospital, Kangwon National University School of Medicine, Gangwon-do, Chuncheon, 24341 South Korea; 3 Interdisciplinary Graduate Program in Medical Bigdata Convergence, Kangwon National University, Gangwon-do, Chuncheon, 24341 South Korea; 4 Department of Ophthalmology, Catholic Kwandong University College of Medicine, Incheon, 21388 Korea

**Keywords:** Retinal diseases, Diabetes, Nutrition

## Abstract

We aimed to evaluate the association between the prevalence of diabetic retinopathy (DR) and coffee consumption in a Korean population. This cross-sectional study was based on data from the 2008–2011 Korean National Health and Nutrition Survey. Among 37,753 survey participants, the data of 1350 subjects with type 2 diabetes who underwent DR examination were analyzed. DR was graded using the modified Airlie House classification system. Coffee consumption data were obtained through food frequency questionnaires and categorized into four groups: almost none, < 1 cup/day, 1 cup/day, and ≥ 2 cups/day. The relationship between DR and coffee consumption was evaluated using multivariable logistic regression models adjusted for age, sex, education, occupation, income, smoking, alcohol intake, body mass index, physical activity, hypertension, dyslipidemia, diabetes duration, and glycated hemoglobin. The prevalence of DR was 20.0%. Non-proliferative DR was observed in 87.8% of all DR patients, and proliferative DR in 12.2%. The prevalence of DR and vision-threatening DR showed a significantly decreasing tendency according to daily coffee consumption (*P* for trend 0.025 and 0.005, respectively) after adjustment for possible confounders. This tendency was more prominent in those aged < 65 years (*P* for trend 0.005 and 0.003, respectively). Our findings suggest coffee consumption might be associated with DR reduction especially in Koreans with diabetes mellitus aged < 65 years.

## Introduction

Diabetic retinopathy (DR) is an important complication of diabetes mellitus (DM) and is a major cause of vision impairment and blindness. DR can significantly affect an individual’s quality of life^1^. The prevalence of DR among diabetic patients varies across countries, ranging from 18% in India to 40% in the United State^2^. Given the global burden of diabetes, there is a great deal of interest in developing nutritional and dietary approaches to reduce or prevent diabetic complications^3–5^.

Coffee is one of the most consumed beverages worldwide^6^. Over the past few decades, many studies have investigated the associations between coffee consumption and various diseases, including cancer^7^, Alzheimer's disease^8^, cardiovascular disease^9^, and type 2 diabetes^10^. Numerous studies including several meta-analyses have indicated that coffee consumption lowers the risk of type 2 diabetes^5,11,12^.

Despite the great interest in the relationship between coffee consumption and diabetes, few studies have evaluated the association between DR and coffee consumption. Some animal studies have suggested that ingestion of coffee might be effective in preventing DR^13–15^; however, the only study conducted in humans showed no significant association between intake of coffee and DR^16^. Therefore, the purpose of this study was to assess the relationship between the prevalence of DR and coffee consumption in a large population.

## Results

**Figure 1:**
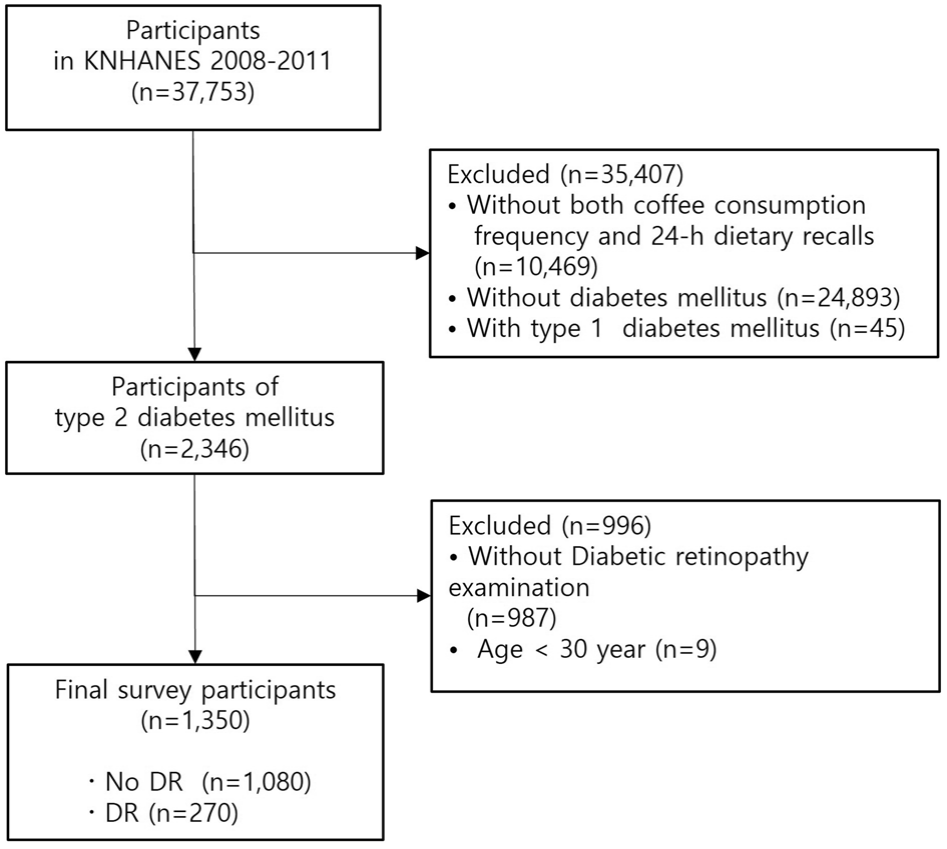
Flowchart of the study participants diagnosed with type 2 diabetes who underwent diabetic retinopathy examination. (KNHANES: Korea National Health and Nutrition Examination Survey).

Among 37,753 survey participants, the data of 1350 participants diagnosed with type 2 diabetes who underwent DR examination were analyzed (Fig. 1). The basic characteristics of the participants are presented in Table 1. The prevalence of any DR and vision-threatening DR (VTDR) in this population was 20.0% and 5.3%, respectively. Among the participants with DR, 87.8% had NPDR and 12.2% had PDR. Compared to participants without DR, the DR group had a significantly higher HbA1c level, a longer duration of diabetes, and a lower BMI; however, the effects of BMI were no longer significant once they were adjusted for demographic and socioeconomic factors (Supplementary Table S1). The VTDR group exhibited a lower rate of high school or college education, lower BMI, higher HbA1c level, and longer diabetes duration compared with the no VTDR group. Additionally, the baseline characteristics of the participants according to inclusion and exclusion participants are presented in Supplementary Table S2.

**Table 1:** General characteristics of DR in patients with type 2 diabetes (n = 1,350).

	All	No DR	Any DR	*P*	No PDR	PDR	*P*	No VTDR	VTDR	*P*
	(n = 1350)	(n = 1080)	(n = 270)		(n = 1317)	(n = 33)		(n = 1278)	(n = 72)	
Age (years)	58.8 ± 0.4	58.6 ± 0.4	59.9 ± 0.8	0.143	58.8 ± 0.4	59.2 ± 1.6	0.648	58.8 ± 0.4	60.2 ± 1.4	0.332
30–49	180 (22.4)	152 (23.7)	28 (17.1)	0.169	176 (22.6)	4 (12.2)	0.153	173 (23.0)	7 (10.7)	0.051
50–64	529 (43.8)	409 (42.7)	120 (48.2)		511 (43.4)	18 (61.9)		495 (43.1)	34 (57.0)	
≥ 65	641 (33.8)	519 (33.6)	122 (34.7)		630 (34.0)	11 (25.9)		610 (33.9)	31 (32.3)	
Sex, male	657 (55.5)	527 (55.5)	130 (55.4)	0.980	644 (55.7)	13 (46.0)	0.350	623 (55.4)	34 (57.2)	0.789
**Education**										
≤ Elementary school	644 (41.7)	516 (41.1)	128 (44.0)	0.267	626 (41.4)	18 (58.0)	0.372	607 (41.3)	37 (49.4)	0.005
Middle school	218 (16.3)	168 (15.4)	50 (20.0)		212 (16.3)	6 (16.2)		202 (15.6)	16 (29.5)	
High school	320 (27.1)	256 (27.7)	64 (24.5)		314 (27.3)	6 (16.4)		306 (27.6)	14 (15.8)	
≥ College	159 (15.0)	135 (15.8)	24 (11.5)		157 (15.1)	2 (9.4)		156 (15.4)	3 (5.3)	
**Household income**										
Quartile 1 (low)	463 (29.1)	378 (29.0)	85 (29.2)	0.981	454 (29.2)	9 (19.9)	0.543	442 (29.0)	21 (30.1)	0.266
Quartile 2	340 (26.3)	260 (26.1)	80 (27.5)		328 (26.2)	12 (34.0)		314 (25.8)	26 (37.7)	
Quartile 3	283 (23.1)	229 (23.3)	54 (22.2)		278 (23.2)	5 (17.2)		271 (23.5)	12 (15.7)	
Quartile 4 (high)	242 (21.5)	196 (21.5)	46 (21.1)		235 (21.3)	7 (28.9)		230 (21.7)	12 (16.5)	
**Occupation**										
White-collar	105 (11.2)	88 (11.9)	17 (8.2)	0.409	101 (11.2)	4 (14.5)	0.814	101 (11.5)	4 (5.4)	0.263
Blue-collar	532 (44.4)	433 (44.4)	99 (44.5)		520 (44.4)	12 (46.8)		504 (44.0)	28 (52.2)	
Others	702 (44.4)	552 (43.7)	150 (47.2)		686 (44.5)	16 (38.7)		663 (44.5)	39 (42.4)	
Current smoking status	350 (31.3)	276 (31.0)	74 (32.5)	0.718	342 (31.3)	8 (34.0)	0.804	325 (30.7)	25 (44.0)	0.089
**Alcohol**										
Non-drinker	559 (35.3)	439 (34.4)	120 (39.3)	0.443	541 (35.2)	18 (44.1)	0.166	522 (35.0)	37 (41.0)	0.638
Social drinker	623 (48.0)	505 (48.4)	118 (46.2)		610 (47.9)	13 (51.3)		595 (48.1)	28 (46.2)	
Heavy drinker	161 (16.7)	130 (17.2)	31 (14.5)		159 (16.9)	2 (4.6)		154 (16.9)	7 (12.8)	
Walking physical activity	573 (40.0)	458 (40.6)	115 (37.7)	0.505	556 (39.8)	17 (53.1)	0.164	537 (39.7)	36 (45.6)	0.393
Moderate physical activity	149 (10.0)	128 (11.0)	21 (5.8)	0.033	148 (9.9)	1 (11.9)	0.839	145 (10.1)	4 (8.3)	0.734
Aerobic physical activity	202 (15.2)	164 (15.3)	38 (14.7)	0.843	199 (15.3)	3 (8.9)	0.326	194 (15.4)	8 (11.9)	0.542
BMI (kg/m^2^)	25.1 ± 0.1	25.2 ± 0.1	24.3 ± 0.2	< 0.001	25.1 ± 0.1	23.2 ± 0.6	0.064	25.1 ± 0.1	23.4 ± 0.3	< 0.001
BMI < 18.5, underweight	24 (1.5)	17 (1.3)	7 (2.4)	0.036	22(1.5)	2(6.6)	0.150	22 (1.5)	2 (2.5)	0.126
18.5 ≤ BMI < 23.0, normal	374 (26.4)	278 (24.7)	96 (33.8)		362(26.4)	12(29.9)		343 (25.9)	31 (37.8)	
23.0 ≤ BMI < 25.0, overweight	313 (23.2)	248 (23.3)	65 (22.6)		305(23.1)	8(27.6)		298 (23.0)	15 (26.4)	
BMI ≥ 25.0, obese	634 (48.8)	533 (50.7)	101 (41.1)		623(49.1)	11(35.8)		610 (49.6)	24 (33.2)	
HbA1c	7.4 ± 0.1	7.3 ± 0.1	8.2 ± 0.1	< 0.0001	7.4 ± 0.1	8.6 ± 0.4	0.199	7.4 ± 0.1	8.8 ± 0.2	< 0.001
Hypertension	832 (58.3)	670 (58.5)	162 (57.2)	0.754	811 (58.2)	21 (60.8)	0.814	793 (58.9)	33 (54.1)	0.069
Hypercholesterolemia	359 (29.4)	279 (28.3)	80 (33.9)	0.152	350 (29.4)	9 (28.6)	0.938	340 (29.3)	19 (31.1)	0.828
Diabetes duration, years	7.7 ± 0.2	6.9 ± 0.3	10.2 ± 0.5	< 0.001	7.5 ± 0.2	13.9 ± 2.0	0.057	7.3 ± 0.2	13.7 ± 1.3	< 0.001
Energy intake (kcal/day)	1915.9 ± 31.3	1913.4 ± 33.5	1926.3 ± 87.0	0.891	1919.3 ± 31.5	1730.6 ± 197.1	0.339	1925.0 ± 32.3	1735.0 ± 98.3	0.065
**Coffee consumption**										
Almost none	231 (15.2)	178 (14.9)	53 (16.5)	0.376	223 (15.1)	8 (19.5)	0.226	217 (15.3)	14 (13.6)	0.252
< 1 time/day	310 (22.1)	249 (21.6)	61 (24.6)		301 (22.1)	9 (24.5)		292 (21.8)	18 (29.5)	
1 time/day	365 (27.7)	293 (27.2)	72 (29.6)		356 (27.4)	9 (39.4)		342 (27.4)	23 (33.8)	
≥ 2 times/day	444 (35.0)	360 (36.3)	84 (29.3)		437 (35.3)	7 (16.6)		427 (35.6)	17 (23.1)	

Data are expressed as means ± standard errors for continuous variables or numbers (proportions) for categorical variables. *P* values were based on the Wilcoxon rank-sum test for continuous variables and the chi-square test for categorical variables.

DR, diabetic retinopathy; BMI, body mass index; HbA1c, glycated hemoglobin; PDR, proliferative diabetic retinopathy; VTDR, vision-threatening diabetic retinopathy.

Patient characteristics compared in terms of the amount of coffee consumption are presented in Supplementary Table S3. The variables with significant differences between different coffee consumption groups included age (*P* < 0.001), sex (*P* < 0.001), education level (*P* < 0.001), household income (*P* < 0.001), occupation (*P* < 0.001), current smoking status (*P* < 0.001), alcohol drinking (*P* < 0.001), walking physical activity (*P* < 0.001), prevalence of hypertension (*P* = 0.002), Energy intake (*P* < 0.001), and BMI (*P* = 0.012). The average age of the group who rarely drank coffee was 61.5 ± 1.1, and that of the group who drank ≥ 2 cups/day was 56.3 ± 0.6. The proportion of men in the group who rarely drank coffee was 41.0%, and that in the group who drank ≥ 2 cups/day was 71.4%. In the group who rarely drink coffee, 9.9% had a college education or higher compared to the 23.3% in the group that drank ≥ 2 cups/day. In the group that rarely drink coffee, 10.7% had a household income above the 4th quartile, and 27.9% in the group who drank ≥ 2 cups/day. The current smoking rate was 19.7% in the group that rarely drank coffee, and 44.9% in the group that drank ≥ 2 cups/day. The prevalence of hypertension was 70.9% in the group that rarely drank coffee, and 51.8% in the group that drank ≥ 2 cups/day. Finally, the average BMI was 24.7 ± 0.2 kg/m^2^ for those who rarely drank coffee, and 25.5 ± 0.2 kg/m^2^ for those who drank ≥ 2 cups/day.

After adjusting for potential confounders, coffee consumption was found to be inversely correlated with the prevalence of any DR and VTDR (*P* for trend = 0.025 for any DR, *P* for trend = 0.005 for VTDR; Table 2). Participants who consumed ≥ 2 cups of coffee per day had lower odds of having any DR (odds ratio [OR] 0.53, 95% confidence interval [CI] 0.28–0.99) and VTDR (OR: 0.30, 95% CI 0.10–0.91) than those who drank almost none after adjustment. The OR for the 1 cup/day group was not statistically significant in the any DR or the VTDR groups. There were no significant correlations between the prevalence of PDR and coffee consumption.

**Table 2:** The prevalence of DR by frequency of coffee consumption from the food frequency questionnaire among participants with type 2 diabetes (n = 1350).

	Coffee consumption				
	Almost none (n = 231)	< 1 cup/day (n = 310)	1 cup/day (n = 365)	≥ 2 cups/day (n = 444)	*P* for trend
**Any DR**					
Case	53	61	72	84	
Crude OR (95% CI)	1.00 (ref.)	1.03 (0.63–1.67)	0.98 (0.61–1.59)	0.73 (0.45–1.18)	0.133
Age and sex adjusted OR (95% CI)	1.00 (ref.)	1.04 (0.64–1.68)	0.99 (0.61–1.60)	0.74 (0.45–1.21)	0.165
Multivariable adjusted OR (95% CI)	1.00 (ref.)	0.95 (0.54–1.68)	0.67 (0.36–1.24)	0.53 (0.28–0.99)	0.025
**VTDR**					
Case	14	18	23	17	
Crude OR (95% CI)	1.00 (ref.)	1.52 (0.68–3.40)	1.39 (0.64–2.99)	0.73 (0.30–1.75)	0.238
Age and sex adjusted OR (95% CI)	1.00 (ref.)	1.53 (0.69–3.38)	1.38 (0.64–2.98)	0.71 (0.30–1.69)	0.235
Multivariable adjusted OR (95% CI)	1.00 (ref.)	1.44 (0.60–3.43)	0.57 (0.21–1.53)	0.30 (0.10–0.91)	0.005
**PDR**					
Case	8	9	9	7	
Crude OR (95% CI)	1.00 (ref.)	0.86 (0.30–2.48)	1.11 (0.38–3.31)	0.37 (0.12–1.13)	0.086
Age and sex adjusted OR (95% CI)	1.00 (ref.)	0.86 (0.30–2.48)	1.13 (0.40–3.23)	0.38 (0.12–1.20)	0.103
Multivariable adjusted OR (95% CI)	1.00 (ref.)	0.73 (0.20–2.60)	0.41 (0.10–1.67)	0.28 (0.06–1.42)	0.071

Multivariable adjustments included age, gender, education, occupation, income, body mass index, energy intake, hypertension, dyslipidemia, duration of diabetes, glycated hemoglobin (%), smoking, drinking, and physical activity (aerobic, moderate, walking level).

DR, diabetic retinopathy; PDR, proliferative diabetic retinopathy; VTDR, vision-threatening diabetic retinopathy; OR, odds ratio; CI, confidence interval.

**Figure 2:**
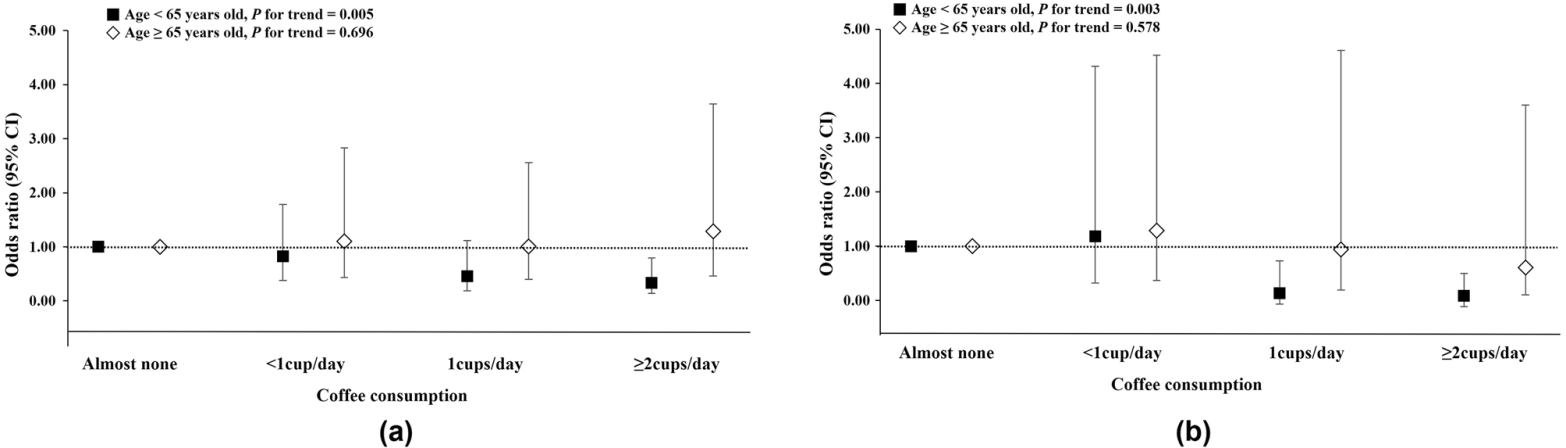
Prevalence of diabetic retinopathy (DR) in terms of coffee consumed among patients with type 2 diabetes by age. Odds ratio of (**a**) any DR and (**b**) vision-threatening diabetic retinopathy (VTDR). The prevalence of any DR and VTDR significantly lowered with higher coffee consumption in all participants (*P* for trend = 0.025 and 0.005, respectively). When the population was divided into two groups according to age, the trend was significant in participants aged < 65 years (*P* for trend = 0.005 and 0.003, respectively) but not in those aged ≥ 65 years.

When the population was divided into two groups according to age, this decreasing tendency of DR according to coffee consumption became more pronounced among participants aged under 65 years (Fig. 2). The overall trend of the inverse relationship between coffee consumption and the prevalence of any DR and VTDR became more significant after adjustment (*P* for trend = 0.005 and 0.003, respectively). However, these trends were not seen in participants aged ≥ 65 years.

We performed a comparison of demographics between the two groups (< 65 vs. ≥ 65 years). The ≥ 65-years-old group had a longer duration of diabetes, a higher rate of hypertension, and a higher proportion of participants who drank < 1 cup of coffee per day. This group had a lower proportion of men, a lower education level, a lower income level, a lower consumption of alcohol and cigarettes, a lower BMI, and a lower HbA1c (Supplementary Table S4).

Next, we analyzed the correlation between the prevalence of DR and the type of coffee consumed (Table 3). Individuals who drank black coffee had lower odds of DR (*P* for trend = 0.040). This tendency was the same in the group who consumed coffee with sugar or cream (*P* for trend = 0.031).

**Table 3:** DR according to the tertiles of coffee intake consumed from one-day 24-h dietary recall among patients with type 2 diabetes (n = 1350).

	Tertile for black coffee intake				Tertile for coffee with sugar or cream intake			
	T1 (low)	T2	T3 (high)	*P* for trend	T1(low)	T2	T3(high)	*P* for trend
Intake (cup/day)	None	≤ 1cup/day	> 1cup/day		None	≤ 1cup/day	> 1cup/day	
**Any DR**								
Case/at risk	193/933	27/137	50/280		190/921	46/251	34/178	
Crude OR (95% CI)	1.00 (ref.)	0.93 (0.53–1.73)	0.77 (0.51–1.65)	0.233	1.00 (ref.)	0.99 (0.62–1.59)	0.85 (0.50–1.42)	0.559
Multivariable adjusted OR (95% CI)	1.00 (ref.)	0.90 (0.45–1.79)	0.57 (0.34–0.95)	0.040	1.00 (ref.)	0.63 (0.35–1.13)	0.55 (0.29–1.06)	0.031

Multivariable adjustments included age, gender, education, occupation, income, body mass index, energy intake, hypertension, dyslipidemia, duration of diabetes, glycated hemoglobin (%), smoking, drinking, and physical activity (aerobic, moderate, walking level).

DR, diabetic retinopathy; the black coffee intake group and coffee with sugar or cream intake group were mutually adjusted; OR, odds ratio; CI, confidence interval.

## Discussion

In this study, we found that participants who reported drinking ≥ 2 cups of coffee per day had a lower prevalence of any DR and VTDR compared with those who reported drinking less than 1 cup of coffee per day. Moreover, we found a negative correlation between the degree of coffee consumption and the prevalence of any DR and VTDR by trend analysis. We also observed that, regardless of the type of coffee, the prevalence of DR tended to decrease with increases in coffee intake of both black coffee and coffee with sugar or cream, adjusted for confounders such as energy intake.

Many studies have shown the inverse association between coffee drinking and the risk of type 2 diabetes^4–6,17^. Carlström et al. reviewed 30 articles about the relationship between coffee and type 2 diabetes and performed a systematic review and meta-analysis^17^. Based on available evidence, they concluded that coffee consumption is inversely associated with the risk of type 2 diabetes. The thermogenic, antioxidative, and anti-inflammatory effects of coffee consumption were all suggested as possible mechanisms behind this association.

However, very few studies have analyzed the relationship between coffee intake and DR. Shin et al. showed that chlorogenic acid (CGA) in coffee sufficiently preserved the expression of occludin and decreased vascular endothelial growth factor (VEGF) levels, leading to decreased blood–retinal barrier breakdown and vascular leakage in a diabetic rat model^13^. Jang et al. demonstrated the protective effects of CGA and coffee on retinal degeneration in mice^14,15^. Under hypoxic conditions, pretreatment with CGA prevented cell death in a concentration-dependent manner^14^. Further, coffee metabolites significantly decreased injury to retinal ganglion cells after induced optic nerve crush^15^. These studies showed that coffee consumption may provide health benefits by preventing retinal degeneration^13–15^. A previous report suggested that CGA inhibits retinal neoangiogenesis during DR by impeding high glucose-induced hypoxia-inducible factor 1-alpha-mediated paracrine VEGF expression in microglia cells and by preventing VEGF-induced angiogenesis in retinal endothelial cells^18^. Collectively, these in vivo animal studies suggest that the action of ingredients present in coffee, such as CGA, may mediate the preventative effect of coffee consumption on DR. To the best of our knowledge, only one large population study by Neelam et al.^16^ has evaluated the association between coffee intake and DR and found no significant association. However, this previous study had a smaller sample size (113 DR cases, 240 controls, total sample size of 353) than ours (270 DR cases, 1080 controls, total sample size of 1350), and this lack of adequate power could be the reason for the lack of statistical significance in the previous study. The authors acknowledged this limitation in their paper. In this study, we divided the participants into four groups based on the amount of coffee consumption. As daily coffee intake increased, the prevalence of DR decreased. However, our study is a cross-sectional study, and therefore it cannot clearly explain the causal relationship between the lower prevalence of DR seen with increased coffee consumption. Thus, cohort studies with a prospective design will be required to fully elucidate the causal relationship.

In this study, participants who drank more coffee tended to have a higher BMI, and BMI was significantly lower in participants with DR than in those without DR. Some studies have reported an association between a higher BMI and a lower prevalence of DR^19,20^. However, other studies reported no association or a positive association with DR^21,22^. Most results showing an inverse association between obesity and DR were obtained in Asian study participants^23^; therefore, ethnic differences are a possible explanation for inconsistency between obesity and DR. Unlike the relatively clear association between higher BMI and increased risk of diabetes, the association between BMI and DR is disputed, due to the conflicting results of previous studies^20,22^. In this study, participants who drank more coffee had a higher BMI and a lower prevalence of DR; however, the association between BMI and DR was not statistically significant (Supplementary Table S1) and it should not be overlooked that BMI remains an important risk factor for the development of DM. Further longitudinal cohort studies are required to clarify whether higher BMI lowers the incidence of DR.

In Korea, the middle aged and older populations drink more coffee with sugar or creamer than any other age group^24^, but the daily total sugar intake was 61.4 g^25^, which is considerably lower than that in the United States (116.4 g)^26^. Although no clear results have been reported with creamers or sugars, it should not be overlooked that excessive intake of saturated fat or simple sugars in cream or sugar can lead to weight gain and insulin resistance^27^.

In this study, the association between coffee consumption and DR was only significant among participants under 65 years of age. There are a few possible reasons for this. First, the duration of diabetes was longer in the ≥ 65-years-old group, and the effect of the duration of diabetes on DR may have been too strong to allow other factors, such as consumption of coffee, to have an impact on DR. Second, it is more likely that the diet pattern, including coffee intake, changed after being diagnosed with diabetes among participants ≥ 65 years old. This assumption is supported by the fact that the ≥ 65-years-old group consumed less alcohol and cigarettes, had a lower BMI and HbA1c, and had a higher proportion of participants who consumed less than one cup of coffee per day than the group aged < 65 years. In particular, the lower HbA1c may be a result of more thorough glycemic control than in the < 65-years-old group and supports our theory of behavioral and dietary changes. However, because this was a cross-sectional study, it was not possible to confirm the change in coffee consumption patterns in patients with our data set. Finally, in the ≥ 65-years-old group, potential people with severe disease may have been excluded due to death, resulting in a selective survival bias.

Our study has several limitations. First, due to the cross-sectional nature of the analysis performed using a predesigned survey, we were not able to define the causal relationship between DR and coffee consumption. Additionally, although we found a negative correlation between the degree of coffee consumption and the prevalence of DR, our results might be affected by “collider bias” because coffee consumption would be associated with both DR and DM^28^. Second, the exact mechanism by which coffee components affect the progression of DR remains unknown. Further studies should be conducted to elucidate this mechanism. Third, the number of participants with DR was small. There were no significant correlations between the prevalence of PDR and coffee consumption, despite coffee intake being inversely correlated with the prevalence of any DR or VTDR. This may be because there were too few patients with PDR to produce statistically significant results. Nevertheless, this study used a nationwide, stratified, multistage, clustered sampling, and standardized assessment methods of seven standardized photographs. To our knowledge, this is the first study to demonstrate the relationship between coffee intake and DR in a large survey analysis.

In conclusion, this study showed that coffee intake inversely correlated with the prevalence of DR in Koreans with DM under 65 years of age, suggesting that coffee consumption might be associated with a reduction in DR. Cohort studies are warranted to fully elucidate the cross-sectional association between coffee consumption and DR.

## Materials and methods

### Study population

This study was based on data acquired from the Korean National Health and Nutritional Examination Survey (KNHANES), which has been conducted by the Division of Health and Nutritional Survey, Korean Centers for Disease Control and Prevention (KCDC) since 1998^29^. It is a cross-sectional, population-based, nationally representative ongoing survey. A multi-stage, stratified, and clustered probability design was used to choose a sample of civilian, non-institutionalized Korean adults. The KNHANES contains three components: a health interview, nutrition survey, and health examination. Data were assembled via household interviews and standardized physical examinations conducted at mobile examination centers (Supplementary Table S5) ^29^.

Data from the Fourth and Fifth KNHANES conducted from 2008 to 2011 (KNHANES IV-2,3 and V-1) were used. A 5-year ophthalmic survey designed by the Korean Ophthalmological Society was conducted between July 2008 and December 2011. Detailed survey information has been previously published^29,30^.

A total of 37,753 patients were surveyed between 2008 and 2011. Participants aged 30–79 years with type 2 diabetes were eligible for inclusion in the present study. Participants aged 30–79 years with type 2 diabetes were eligible for inclusion in the present study. We excluded participants with missing data of both FFQs and 24-h dietary recalls (n = 10,469), without type 2 diabetes (n = 24,893), and with type 1 diabetes (n = 45). Furthermore, we excluded participants who did not have accessible data regarding fundus photographs in the diabetic retinopathy examination (n = 987) or were younger than 30 years of age (n = 9). A total of 1,350 participants were enrolled and included in the final analysis (Fig. 1). This survey was reviewed and approved by the Institutional Review Boards (IRBs) of the Korean Centers for Disease Control and Prevention (IRB numbers: 2008-04EXP-01-c, 2009-01CON-03-2C, 2010-02CON-21-C, 2011-02CON-06-C) and written informed consent was provided by all participants. All experiments and examinations were performed in accordance with relevant guidelines and regulations.

### Evaluation of diabetes

Diabetes was defined as a diagnosis of diabetes, the use of oral hypoglycemic medications or insulin, and/or a fasting blood glucose level ≥ 126 mg/dL. Participants aged younger than 30 years at the time of diagnosis were considered to have type 1 diabetes and were excluded.

### Evaluation of DR

In cooperation with the KCDC, eye examinations were conducted by ophthalmologists from the Korean Ophthalmologic Society. Non-mydriatic 45° digital fundus photography (TRC-NW6S; Topcon, Tokyo, Japan) was performed in all participants ≥ 19 years old. For each participant, one 45° nonmydriatic digital retinal image centered on the fovea was taken per eye (two images per person). In participants who had a history of diabetes, random blood glucose level ≥ 200 mg/dL, or doubtful DR findings in the non-mydriatic fundus photographs, seven standard photographs from the Early Treatment for Diabetic Retinopathy Study (ETDRS) were obtained from both eyes after pharmacologic pupil dilatation with the same Non-mydriatic 45° digital fundus photography (TRC-NW6S; Topcon, Tokyo, Japan)^31^. All 1350 final participants with type 2 diabetes received seven standard fundus photographs from the ETDRS.

DR was defined if any of the following characteristic lesions were present, based on the Early Treatment for Diabetic Retinopathy Study severity scale: microaneurysms, hemorrhages, cotton wool spots, intraretinal microvascular abnormalities, hard exudates, venous beading, or new vessels. Each eye of participants was assigned a DR severity score according to the modified Airlie House Classification system^31^; detailed grading information has been previously published^19^. The level of DR was measured depending on the worse eye. Eyes were graded as either no DR (levels 10–13) or any DR (levels 14–80). DR was divided further into minimal non-proliferative DR (NPDR, levels 14–20), mild NPDR (level 31), moderate NPDR (levels 41–47), severe NPDR (level 51), and proliferative DR (level > 60). Any DR was further subdivided into non-proliferative DR (NPDR, levels 14–51), and proliferative DR (PDR, level > 60).

Macular edema (ME) was defined by hard exudates within one disk diameter from the foveal center in the presence of blot hemorrhage and microaneurysms or by the existence of focal photocoagulation scars in the macular area. Vision-threatening DR (VTDR) was determined by the presence of severe NPDR, PDR, or clinically-significant ME^31^.

### Coffee consumption

Dietary information was collected by trained dietitians through a 63-item non-quantitative food frequency questionnaire (FFQ) over the past year and through a one-day 24-h dietary recall. The overall frequency of coffee consumption in the past year was obtained from a non-quantitative food frequency questionnaire (FFQ). Participants were asked to report how frequently they consumed coffee over the previous year based on 10 categories: 3 cups a day, 2 cups a day, 1 cup a day, 4–6 cups a week, 2–3 cups a week, 1 cup a week, 2–3 cups a month, 1 cup a month, 6–11 cups a year, and almost none. The frequency of coffee consumption was categorized into the following four groups: almost none (6–11 cups a year, and almost none), < 1 cup a day (4–6 cups a week, 2–3 cups a week, 1 cup a week, 2–3 cups a month, 1 cup a month), 1 cup a day, and ≥ 2 cups a day^32^. Information on types and amount of coffee calculated from the 24-h dietary recalls was collected from the same participants who completed the FFQs. We quantified the intake (serving or gram) of specific coffee types such as black coffee or coffee with sugar and powder creamer from a single 24-h dietary recalls, because the FFQ did not include the consumption by type of coffee. The quantitative 24-h dietary recall questionnaire collected detailed quantitative information on all foods and beverages consumed in the past 24 h (time, location, type of food, amount, cooking method) using open-ended dietary assessment methods. Using data from 24-h dietary recalls, we categorized types of coffee as “black coffee” and “coffee with sugar or powder creamer,” and the intake of coffee type was divided into three groups as follows: none, ≤ 1 cup a day, and > 1 cup a day.

### Assessment of other variables

Covariates for the statistical models were assessed based on related studies^33–36^. Potential confounders included age; sex; health-related behaviors such as smoking, alcohol use, and physical activity; socioeconomic status including education, occupation, and household income; and comorbid medical conditions, such as hypertension, hypercholesterolemia, glycated hemoglobin (HbA1c) level, body mass index (BMI), and diabetes duration. Information regarding demographic and social factors was collected using a standardized questionnaire during a health interview. “Current smoker” was defined as currently smoking with a smoking history of 100 or more cigarettes in the participant’s lifetime. The participants were divided into three groups based on their level of alcohol consumption: non-drinker, social drinker, and heavy drinker. The Korean National Nutrition Survey defined the high-risk drinking rate as an average drinking rate of seven or more glasses (five glasses for women) per drinking session, with a frequency of drinking more than twice a week. Physical activity was categorized into the following independent categories: aerobic, moderate, and walking. Aerobic activity was defined as physical activity for least 2 h and 30 min/week, medium- or high-intensity physical activity for 1 h and 15 min, or both medium- and high-intensity physical activity. Moderate physical activity was defined as participation in at least five days of ≥ 30 min/day of less intensive activity. Participants performing at least five days of ≥ 30 min/day of walking were classified into the walking physical activity group. Educational level was categorized into following four groups: elementary school graduate or less, middle school graduate, high school graduate, and college graduate or above. The participants were stratified into four quartiles according to their equivalent household income (quartile 1, lowest; quartile 4, highest). Occupation was categorized into the following three groups: white collar, blue collar, and others.

Blood pressure (BP) was measured using standard methods with the patient in a sitting position. Three measurements were taken in all participants at 5-min intervals, and the average of the second and third measurements was used in the analysis. Hypertension was defined as a systolic BP > 140 mmHg and diastolic BP > 90 mmHg, or if the individual had been prescribed antihypertensive medication. Blood samples were collected in the morning after fasting for at least 8 h. Fasting glucose, HbA1c, and total cholesterol levels were measured at a certified laboratory. Hypercholesterolemia was defined as a total cholesterol concentration > 240 mg/dL or if the individual had been prescribed cholesterol-lowering medication. Diabetes duration was determined as the difference between the current age of the patient and the age at diabetes onset.

### Statistical analysis

The demographic and clinical characteristics of the study participants are presented as means (standard error) or number (proportion). Differences between proportions were tested using the chi-square test and differences in continuous variables were tested using the Wilcoxon rank-sum test.

Logistic regression models were used to investigate the association between coffee consumption and the prevalence of any DR, PDR, and VTDR. The first logistic regression model was adjusted for age and sex. A second model was adjusted for other potential confounders, including education, occupation, income, smoking, alcohol intake, BMI, physical activity, energy intake, hypertension, dyslipidemia, duration of diabetes, and HbA1c (%). Moreover, we assessed the association of coffee without sugar or cream and coffee with sugar or cream. If participants drank both types of coffee, the association was assessed after adjusting for the other type of coffee. To test for a linear trend across increasing amounts of coffee consumption, we modeled categories of coffee consumption as a continuous variable. In addition, we created subgroups divided by age group (< 65 and ≥ 65 years) and examined associations between subgroups and diagnosis of any DR, PDR, and VTDR.

Statistical analyses were performed using SAS software version 9.4 (SAS Institute Inc., Cary, NC, USA). All statistical analyses accounted for the KNHANES’s complex sample design and weighting. Statistical significance was set at *P* < 0.05.

## Supplementary Information

Supplementary Information.

## Data Availability

The datasets used and/or analyzed during the current study are available from the corresponding author on reasonable request.
