# Supplementary material for: Coffee consumption and diabetic retinopathy in adults with diabetes mellitus
Source: Sci Rep. 2022 Mar 3;12:3547. doi: 10.1038/s41598-022-07192-6 (PMC8894381; doi:10.1038/s41598-022-07192-6)
Supplement: Supplementary file 1 — Supplementary Information. [file 41598_2022_7192_MOESM1_ESM.docx]

**Coffee Consumption and Diabetic Retinopathy in Adults with Diabetes Mellitus**

Hak Jun Lee^1,†^, Ji In Park^2,†^, Sung Ok Kwon^3^, Daniel Duck-Jin Hwang^1,4,*^

^1^Department of Ophthalmology, Hangil Eye Hospital, Incheon 21388, Korea

^2^Department of Medicine, Kangwon National University Hospital, Kangwon National University School of Medicine, Chuncheon 24341, Gangwon-do, South Korea

^3^Interdisciplinary Graduate Program in Medical Bigdata Convergence, Kangwon National University, Chuncheon 24341, Gangwon-do, South Korea

^4^Department of Ophthalmology, Catholic Kwandong University College of Medicine, Incheon 21388, Korea

**Supplementary Table S1.** The adjusted odds ratio of DR by possible confounders among participants with type 2 diabetes (n = 1,350).

|  | Adjusted OR (95% CI) | *P* |
| --- | --- | --- |
| Age (years) |  |  |
| 30–49 | 1.00 (ref.) |  |
| 50–64 | 0.78(0.39-1.56) | 0.475 |
| ≥65 | 0.71(0.33-1.53) | 0.384 |
| *P* for trend | 0.302 |  |
| Sex, male, n (%) | 0.96(0.58-1.61) | 0.882 |
| Education, n (%) |  |  |
| ≤Elementary school | 1.00 (ref.) |  |
| Middle school | 1.20(0.70-2.08) | 0.507 |
| High school | 1.01(0.56-1.81) | 0.985 |
| ≥College | 0.90(0.36-2.27) | 0.823 |
| *P* for trend | 0.648 |  |
| Household income (%) |  |  |
| Quartile 1 (low) | 1.00 (ref.) |  |
| Quartile 2 | 1.11(0.64-1.91) | 0.711 |
| Quartile 3 | 1.09(0.56-2.13) | 0.797 |
| Quartile 4 (high) | 0.99(0.48-2.06) | 0.983 |
| *P* for trend | 0.982 |  |
| Occupation |  |  |
| White-collar | 1.00 (ref.) |  |
| Blue-collar | 1.38(0.51-3.74) | 0.528 |
| Others | 1.31(0.48-3.59) | 0.594 |
| Current smoking status (yes %) | 0.84(0.47-1.52) | 0.567 |
| Alcohol (%) |  |  |
| Non-drinker | 1.00 (ref.) |  |
| Social drinker | 0.80(0.52-1.23) | 0.311 |
| Heavy drinker | 0.72(0.34-1.53) | 0.387 |
| *P* for trend | 0.302 |  |
| Walking physical activity (yes %) | 0.78(0.52-1.17) | 0.229 |
| Moderate physical activity (yes %) | 0.45(0.22-0.91) | 0.026 |
| Aerobic physical activity (yes %) | 1.19(0.68-2.09) | 0.540 |
| BMI (kg/m2) |  |  |
| BMI <18.5, underweight | 0.62(0.17-2.21) | 0.455 |
| 18.5≤ BMI <23.0, normal | 1.00 (ref.) |  |
| 23.0≤ BMI <25.0, overweight | 0.86(0.52-1.41) | 0.546 |
| BMI≥25.0, obese | 0.72(0.44-1.17) | 0.184 |
| HbA1c (%) | 1.45(1.27-1.66) | <.0001 |
| Hypertension (yes %) | 1.17(0.78-1.74) | 0.449 |
| Hypercholesterolemia (yes %) | 1.18(0.76-1.83) | 0.452 |
| Diabetes duration, years | 2.30(1.57-3.38) | <.0001 |
| Energy intake (kcal/day) |  |  |
| Quintile 1 | 1.00 (ref.) |  |
| Quintile 2 | 0.62(0.36-1.09) | 0.097 |
| Quintile 3 | 0.67(0.36-1.23) | 0.197 |
| Quintile 4 | 0.75(0.40-1.39) | 0.357 |
| *P* for trend | 0.529 |  |

**Supplementary Table S2.** General characteristics according to inclusion and exclusion participants in The Korea National Health and Nutrition Examination Survey (KNHANES)

|  | **Inclusion** | **Exclusion, ≥30 years** | **Exclusion, all** | ***P for***  ***inclusion vs.***  ***exclusion***  ***(≥30 years)*** | ***P for inclusion vs. exclusion***  ***(all)*** |
| --- | --- | --- | --- | --- | --- |
|  | (n=1350) | (n=24,679) | (n=36,403) |  |  |
| Age (years) | 58.8±0.4 | 49.5±0.2 | 36.8±0.2 | <.001 | <.001 |
| ≤19 | - | - | 9682(26.6) | <.001 | <.001 |
| 20-29 | - | - | 3392(9.3) |  |  |
| 30–49 | 180 (22.4) | 10817(46.4) | 10817(29.7) |  |  |
| 50–64 | 529 (43.8) | 6783(29.1) | 6783(18.6) |  |  |
| ≥65 | 641 (33.8) | 5729(24.6) | 5729(15.7) |  |  |
| Sex, male | 657 (55.5) | 10056(43.1) | 16538(45.4) | <.001 | 0.019 |
| Education |  |  |  |  |  |
| ≤Elementary school | 644 (41.7) | 6594(23.3) | 14148(31.7) | <.001 | <.001 |
| Middle school | 218 (16.3) | 2675(12.2) | 3939(12.3) |  |  |
| High school | 320 (27.1) | 6788(34.5) | 8858(31.4) |  |  |
| ≥College | 159 (15.0) | 5757(29.9) | 7175(24.6) |  |  |
| Household income |  |  |  |  |  |
| Quartile 1 (low) | 463 (29.1) | 4698(17.3) | 5942(15.3) | <.001 | <.001 |
| Quartile 2 | 340 (26.3) | 5484(26.1) | 8727(26.7) |  |  |
| Quartile 3 | 283 (23.1) | 5831(28.6) | 9866(29.8) |  |  |
| Quartile 4 (high) | 242 (21.5) | 5765(28.0) | 9451(28.3) |  |  |
| Occupation |  |  |  |  |  |
| White-collar | 105 (11.2) | 3942(21.5) | 5007(21.6) | <.001 | <.001 |
| Blue-collar | 532 (44.4) | 9134(43.7) | 10114(38.0) |  |  |
| Others | 702 (44.4) | 8678(34.9) | 11606(40.5) |  |  |
| Current smoking status | 350 (31.3) | 6764(35.8) | 7817(35.6) | <.001 | <.001 |
| Alcohol |  |  |  |  |  |
| Non-drinker | 559 (35.3) | 6678(25.9) | 9512(28.4) | <.001 | <.001 |
| Social drinker | 623 (48.0) | 11832(54.5) | 14627(52.0) |  |  |
| Heavy drinker | 161 (16.7) | 3327(19.7) | 4283(19.6) |  |  |
| Walking physical activity | 573 (40.0) | 8969(40.4) | 10683(42.8) | 0.898 | 0.1398 |
| Moderate physical activity | 149 (10.0) | 2778(12.1) | 3151(11.9) | 0.084 | 0.1117 |
| Aerobic physical activity | 202 (15.2) | 2875(14.1) | 3846(14.7) | 0.451 | 0.8163 |
| BMI (kg/m^2^) | 25.1±0.1 | 23.8±0.0 | 22.7±0.0 | <.001 | <.001 |
| BMI <18.5, underweight | 24 (1.5) | 830(3.5) | 5720(14.1) | <.001 | <.001 |
| 18.5≤ BMI <23.0, normal | 374 (26.4) | 8674(38.5) | 12878(39.7) |  |  |
| 23.0≤ BMI <25.0, overweight | 313 (23.2) | 5401(24.9) | 6472(20.1) |  |  |
| BMI≥25.0, obese | 634 (48.8) | 7109(33.1) | 8293(26.0) |  |  |
| Hypertension | 832 (58.3) | 7372(30.7) | 7540(25.6) | <.001 | <.001 |
| Hypercholesterolemia | 359 (29.4) | 2672(12.1) | 2754(10.1) | <.001 | <.001 |

Data are expressed as means ± standard errors for continuous variables or numbers (proportions) for categorical variables. *P*-values are based on the Wilcoxon rank-sum test for continuous variables and chi-square tests for categorical variables. BMI, body mass index. * physically active: walking ≥5 days/week and ≥30 min/day.

**Supplementary Table S3.** General characteristics according to coffee consumption among patients with type 2 diabetes (n = 1,350).

|  | All | Almost none | <1cup/day | 1cups/day | ≥2cups/day | *P* |
| --- | --- | --- | --- | --- | --- | --- |
| n(%) | 1350(100.0) | 231(15.2) | 310(22.1) | 365(27.7) | 444(35.0) |  |
| Age (yrs) | 58.8±0.4 | 61.5±1.1 | 59.7±0.8 | 59.8±0.7 | 56.3±0.6 | <0.001 |
| 30-49 yr | 180(22.4) | 22(19.4) | 35(19.8) | 41(19.3) | 82(27.8) | 0.001 |
| 50-64 yr | 529(43.8) | 71(34.1) | 113(42.9) | 143(45.1) | 202(47.5) |  |
| ≥ 65 yr | 641(33.8) | 138(46.5) | 162(37.3) | 181(35.6) | 160(24.8) |  |
| Sex (male, %) | 657(55.5) | 77(41.0) | 124(47.0) | 165(50.2) | 291(71.4) | <0.001 |
| Education (%) |  |  |  |  |  |  |
| ≤Elementary school | 644(41.7) | 148(55.7) | 169(50.0) | 185(46.2) | 142(26.7) | <0.001 |
| Middle school | 218(16.3) | 26(11.9) | 43(13.1) | 63(17.1) | 86(19.6) |  |
| High school | 320(27.1) | 40(22.5) | 68(24.2) | 88(27.7) | 124(30.4) |  |
| ≥College | 159(15.0) | 15(9.9) | 30(12.7) | 26(9.1) | 88(23.3) |  |
| Household income (%) |  |  |  |  |  |  |
| Quartile 1 (low) | 463(29.1) | 99(37.9) | 125(37.9) | 125(28.2) | 114(20.4) | <0.001 |
| Quartile 2 | 340(26.3) | 68(28.3) | 77(26.2) | 87(25.8) | 108(26.0) |  |
| Quartile 3 | 283(23.1) | 40(23.2) | 57(19.5) | 78(22.7) | 108(25.7) |  |
| Quartile 4 (high) | 242(21.5) | 21(10.7) | 43(16.4) | 71(23.3) | 107(27.9) |  |
| Occupation (%) |  |  |  |  |  |  |
| White-collar | 105(11.2) | 9(9.3) | 10(4.9) | 29(10.0) | 57(17.1) | <0.001 |
| Blue-collar | 532(44.4) | 74(31.6) | 112(46.5) | 134(38.8) | 212(53.2) |  |
| Others | 702(44.4) | 146(59.2) | 188(48.6) | 200(51.2) | 168(29.7) |  |
| Current smoking status (%) | 350(31.3) | 34(19.7) | 51(18.1) | 88(31.2) | 177(44.9) | <0.001 |
| Alcohol (%) |  |  |  |  |  |  |
| Non-drinker | 559(35.3) | 152(59.2) | 141(41.1) | 140(32.4) | 126(23.6) | <0.001 |
| Social drinker | 623(48.0) | 55(24.6) | 136(43.7) | 186(54.3) | 246(55.8) |  |
| Heavy drinker | 161(16.7) | 22(16.2) | 32(15.2) | 38(13.3) | 69(20.6) |  |
| Walking physical activity (%) | 573(40.0) | 114(49.3) | 127(40.6) | 159(41.7) | 173(34.3) | 0.021 |
| Moderate physical activity (%) | 149(10.0) | 24(10.0) | 42(13.1) | 43(9.0) | 40(8.8) | 0.410 |
| Aerobic physical activity (%) | 179(14.4) | 25(14.7) | 32(11.0) | 64(16.8) | 81(16.9) | 0.295 |
| BMI (kg/m^2^) | 25.1±0.1 | 24.7±0.2 | 24.6±0.2 | 25.0±0.3 | 25.5±0.2 | 0.012 |
| BMI <18.5, Underweight | 24(1.5) | 5(1.7) | 6(2.5) | 10(2.3) | 3(0.3) | 0.101 |
| 18.5≤ BMI <23.0, normal | 374(26.4) | 73(29.7) | 89(30.3) | 103(25.8) | 109(23.1) |  |
| 23.0≤ BMI <25.0, overweight | 313(23.2) | 52(26.2) | 73(23.5) | 83(23.6) | 105(21.4) |  |
| BMI≥25.0, obese | 634(48.8) | 101(42.4) | 140(43.7) | 168(48.3) | 225(55.2) |  |
| HbA1c (%) | 7.4±0.1 | 7.3±0.2 | 7.3±0.1 | 7.5±0.1 | 7.5±0.1 | 0.397 |
| Hypertension (%) | 832(58.3) | 168(70.9) | 199(61.3) | 214(57.1) | 251(51.8) | 0.002 |
| Hyper-cholesterolemia (%) | 359(29.4) | 58(31.9) | 84(28.0) | 94(28.4) | 123(30.0) | 0.880 |
| Diabetes duration (yrs) | 7.7±0.2 | 7.8±0.5 | 7.9±0.5 | 7.7±0.5 | 7.4±0.4 | 0.889 |
| Energy intake (kcal/day) | 1915.9±31.3 | 1709.1±79.2 | 1867.2±59.8 | 1838.3±50.4 | 2098.1±62.3 | < 0.001 |

Data are expressed as means ± standard errors for continuous variables or numbers (proportions) for categorical variables. *P*-values are based on the Kruskal-Wallis test or one-way analysis of variance for continuous variables and chi-square tests for categorical variables. DR, diabetic retinopathy; BMI, body mass index; HbA1c, glycated hemoglobin. * physically active: walking ≥5 days/week and ≥30 min/day

**Supplementary Table S4.** General characteristics according to age group among patients with type 2 diabetes (n=1,350).

|  | **<65 years** | **≥65 years** | ***P*** |
| --- | --- | --- | --- |
| N (%) | 709(66.2) | 642(33.8) |  |
| Sex, male, n (%) | 378(62.9) | 279(41.1) | <.001 |
| Education, n (%) |  |  |  |
| ≤Elementary school | 218(27.1) | 426(70.1) | <.001 |
| Middle school | 135(18.6) | 83(11.7) |  |
| High school | 227(34.4) | 93(12.8) |  |
| ≥College | 122(19.9) | 37(5.4) |  |
| Household income, n (%) |  |  |  |
| Quartile 1 (low) | 149(20.3) | 314(46.6) | <.001 |
| Quartile 2 | 186(26.7) | 154(25.6) |  |
| Quartile 3 | 192(27.2) | 91(15.0) |  |
| Quartile 4 (high) | 174(25.8) | 68(12.7) |  |
| Occupation, n (%) |  |  |  |
| White-collar | 96(16.2) | 9(1.4) | <.001 |
| Blue-collar | 349(52.2) | 183(29.1) |  |
| Others | 255(31.5) | 447(69.4) |  |
| Smoking status, n (%) |  |  |  |
| Never | 361(42.9) | 385(61.2) | <.001 |
| Former | 121(19.2) | 126(20.4) |  |
| Current | 223(37.9) | 127(18.4) |  |
| Alcohol (%) |  |  |  |
| Non-drinker | 208(25.0) | 351(55.5) | <.001 |
| Social drinker | 365(52.0) | 258(40.0) |  |
| Heavy drinker | 132(22.9) | 29(4.5) |  |
| Walking physical activity (%) | 290(38.9) | 283(42.1) | 0.309 |
| Moderate physical activity (%) | 79(10.3) | 70(9.3) | 0.515 |
| Aerobic physical activity (%) | 118(17.1) | 84(11.5) | 0.012 |
| BMI, kg/m^2^ | 25.3±0.2 | 24.6±0.1 | 0.001 |
| BMI < 18.5, underweight | 9(1.2) | 15(2.3) | 0.013 |
| 18.5 ≤ BMI < 23.0, normal | 169(23.5) | 205(32.2) |  |
| 23.0 ≤ BMI < 25.0, overweight | 168(24.2) | 145(21.1) |  |
| BMI ≥ 25.0, obese | 361(51.1) | 273(44.4) |  |
| HbA1c (%) | 7.5±0.1 | 7.2±0.1 | 0.004 |
| Hypertension (%) | 377(51.2) | 455(72.2) | <.001 |
| Hypercholesterolemia (%) | 218(30.5) | 141(26.9) | 0.278 |
| Diabetes duration, years | 6.5±0.3 | 9.5±0.4 | <.001 |
| Energy intake (kcal/day) | 2070.6±42.1 | 1613.5±35.4 | <.001 |
| Coffee consumption |  |  |  |
| Almost none | 93(12.3) | 138(20.9) | <.001 |
| <1cup/day | 148(21.0) | 162(24.4) |  |
| 1cups/day | 184(26.9) | 181(29.1) |  |
| ≥2cups/day | 284(39.8) | 160(25.6) |  |

Data are expressed as means ± standard errors for continuous variables or numbers (proportions) for categorical variables. *P*-values are based on the Wilcoxon rank-sum test for continuous variables and chi-square tests for categorical variables. DR, diabetic retinopathy; BMI, body mass index. * physically active: walking ≥5 days/week and ≥30 min/day

**Supplementary Table S5.** The three survey items of The Korea National Health and Nutrition Examination Survey (KNHANES)

| Investigation | Content |
| --- | --- |
| Health interview | Household survey, household income, education, occupation, smoking, drinking, obesity and weight control, physical activity, morbidity, medical use, vaccination and health examination, activity restrictions and quality of life, accident and addiction, safety awareness, mental health, women’s health, education, and economic status |
| Nutrition survey | Food and nutrient intake from 24hr dietary recall, food frequency questionnaire, dietary behavior, dietary supplements |
| Health examination | Obesity, high blood pressure, diabetes, dyslipidemia, liver disease, kidney disease, anemia, lung disease, oral disease, eye disease |
